# Locating the epileptogenic zone for drug-resistant epilepsy through neuroelectrophysiological brain network topology

**DOI:** 10.3389/fnins.2026.1781032

**Published:** 2026-02-11

**Authors:** Wenzhao Zhang, Zhe Shi, Weixin Kong, Yuanchu Gong, Wentao Lin, Zhijun Wu, Yanming Han, Yaqing Liu, Tiancheng Wang

**Affiliations:** 1 The Second Hospital & Clinical Medical School, Lanzhou University, Lanzhou, China; 2 School of Information Science and Engineering, Lanzhou University, Lanzhou, China; 3 School of Information Science and Technology, Fudan University, Shanghai, China

**Keywords:** brain network topology, drug-resistant epilepsy, epileptogenic zone, machine learning, stereoelectroencephalography

## Abstract

**Introduction:**

Drug-resistant epilepsy (DRE) constitutes approximately one-third of the epilepsy population, posing a significant challenge due to low seizure freedom rates. Accurate localization of the epileptogenic zone (EZ) is the prerequisite for successful surgery. However, the limitations of conventional visual inspection underscore an urgent need for novel localization strategies based on quantitative brain network topology.

**Methods:**

This study established a hierarchical analytical framework to independently analyze neuroelectrophysiological signals from scalp EEG (used for macroscopic hypothesis formulation) and stereoelectroencephalography (SEEG, used for mesoscopic confirmation). We included 25 patients with favorable surgical outcomes and constructed brain networks from ictal and interictal recordings. Subsequently, we evaluated the diagnostic value of these network features using machine learning classifiers [including Support Vector Machine (SVM), Random Forest, etc.].

**Results:**

In SEEG, the EZ exhibited significantly reduced topological metrics (specifically node degree, clustering coefficient, and local efficiency) compared to non-EZ regions (*P* < 0.001), indicating that the epileptogenic focus is a functionally isolated node. The SVM model based on interictal scalp EEG features achieved superior diagnostic performance (AUC = 0.927, Accuracy = 85.7%, Sensitivity = 85.7%, Specificity = 85.7%). In the SEEG modality, we applied Log-transformation and Z-score normalization to overcome individual variations in implantation schemes. This processing significantly boosted the performance of the interictal SEEG model (SVM) (AUC = 0.872, Accuracy = 81.9%, Sensitivity = 83.1%, Specificity = 80.7%).

**Discussion:**

These findings confirm the stability of the EZ's topological signature in the resting state and demonstrate a stepwise workflow: scalp EEG provides coarse localization of the potential EZ to guide SEEG implantation, while SEEG offers more precise surgical recommendations for EZ localization.

## 1 Introduction

Epilepsy remains a pervasive neurological disorder, affecting approximately 50 million people worldwide (World Health Organization, 2019). Over the past few decades, the pharmacological arsenal against epilepsy has expanded significantly, with more than 20 new antiseizure medications (ASMs) introduced. Despite this progress, the proportion of patients with drug-resistant epilepsy (DRE) has remained strikingly constant, affecting up to one-third of the epilepsy population (Klein et al., 2024). In pediatric cohorts, specific etiologies such as focal cortical dysplasia (FCD) further exacerbate these challenges, often leading to severe neurodevelopmental delays (Splitkova et al., 2025). For these patients, surgical interventions, such as resective surgery or stereoelectroencephalography (SEEG)-guided radiofrequency thermocoagulation (RF-TC), offer the most promising potential for seizure freedom (Englot and Chang, 2014; Gonzalez-Martinez, 2025). However, surgical success is heavily dependent on the precise delineation and complete treatment of the epileptogenic zone (EZ) (Guye et al., 2017). A landmark longitudinal study revealed that seizure freedom rates can decline significantly over time, with late recurrence suggesting that current localization methods often fail to fully capture the complex spatiotemporal extent of the epileptogenic substrate (de Tisi et al., 2011).

The traditional clinical gold standard for identifying the EZ relies on the visual inspection of ictal onset patterns in intracranial EEG or SEEG recordings. However, this “focal” perspective is increasingly challenged by the understanding of epilepsy as a network disorder, where seizures arise from abnormal synchronization within distributed “epileptogenic networks” (Guye et al., 2017). Consequently, conventional visual biomarkers, such as the seizure-onset zone (SOZ), are often imperfect surrogates for the EZ, potentially missing critical nodes involved in seizure propagation (Balaji and Parhi, 2022). To address this limitation, recent advancements in signal analysis have shifted toward quantitative characterization of brain networks. Methodologies based on functional connectivity and graph theory have demonstrated that the topological architecture of brain networks can reveal pathological “hubs” and altered information flow, offering biomarkers that are more robust than subjective visual analysis (Wang et al., 2024b,a). Furthermore, recent studies have successfully applied machine learning and deep learning techniques to these network features, achieving promising results in predicting surgical outcomes and localizing epileptogenic tissue (Liu et al., 2025; Li et al., 2025; Wang et al., 2024c).

In current clinical practice, the localization of the EZ follows a hierarchical workflow transitioning from non-invasive to invasive modalities. Initially, scalp EEG is utilized to formulate a hypothesis regarding the epileptogenic focus, which guides the implantation strategy for SEEG electrodes. Subsequently, invasive SEEG recordings provide the high spatiotemporal resolution required to confirm the EZ (Gonzalez-Martinez, 2025). However, a critical gap exists in integrating these two scales. Scalp EEG provides global coverage but suffers from low spatial resolution due to volume conduction (Cohen, 2017). While research efforts have employed electrical source imaging (ESI) to estimate cortical activity and predict outcomes non-invasively (Corona et al., 2023; Lin et al., 2025), these methods still face challenges in validation against ground-truth intracranial data. Conversely, invasive SEEG offers high local precision but is limited by spatial sampling (“tunnel vision”) and cannot capture global network dynamics outside the electrode coverage (Parvizi and Kastner, 2018). Therefore, a comprehensive strategy that combines the macroscopic topology from non-invasive signals and the mesoscopic topology from invasive recordings is essential for improving surgical precision.

Building on this clinical logic, the present study aims to refine EZ localization by employing a hierarchical analysis framework to independently analyze signals from both non-invasive scalp EEG and invasive SEEG. We hypothesize that the EZ exhibits unique, modality-dependent topological signatures that possess diagnostic value. To capture these traits, we analyzed signals across both ictal and interictal states, utilizing ESI to reconstruct cortical activity from scalp EEG and contrasting these findings with direct SEEG recordings. By constructing functional brain networks and extracting key graph theoretic metrics (e.g., Betweenness centrality, clustering coefficient), we quantified the functional distinctions between the EZ and the non-epileptogenic zone (NEZ). Furthermore, we introduced a connectivity density analysis to probe the physiological mechanisms underlying the epileptic network architecture. Finally, leveraging machine learning algorithms to learn these topological features, we established robust classification models at both the EEG and SEEG levels respectively to identify epileptogenic nodes. This approach aims to provide a quantitative, network-based decision support tool to augment the standard clinical workflow for patients with DRE.

## 2 Material and methods

**Figure 1:**
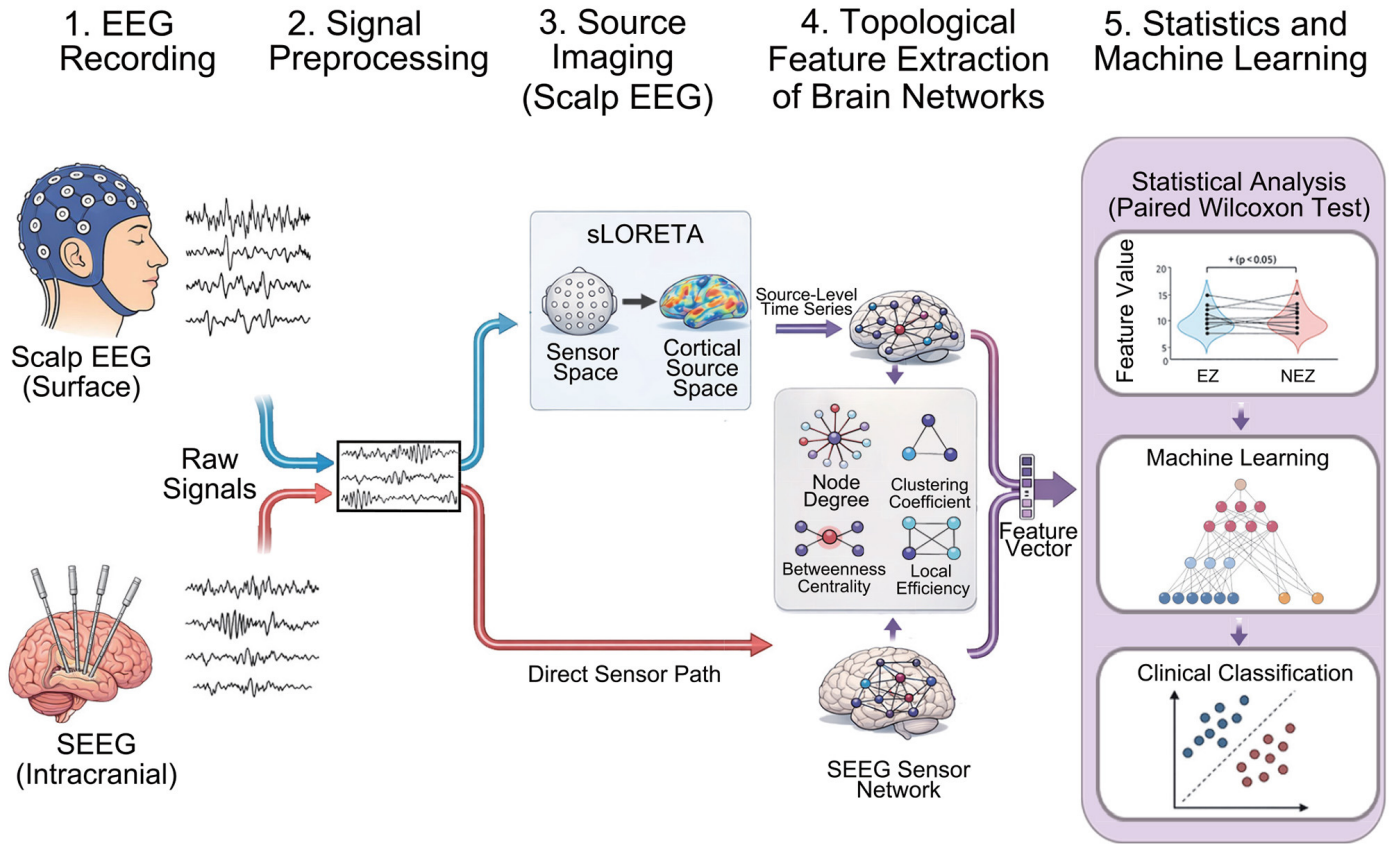
Overall workflow for locating the EZ in DRE.

Our work followed these steps: (1) EEG recording; (2) Signal preprocessing; (3) Source imaging (scalp EEG); (4) Topological feature extraction of brain networks; (5) Statistics and Machine Learning. The details are shown in Figure 1.

### 2.1 Patients

This study included 25 patients who underwent epilepsy surgery at the Second Hospital of Lanzhou University between 2017 and 2023. The inclusion criteria were as follows: (i) having undergone preoperative video electroencephalography (VEEG) and SEEG examinations; (ii) having undergone both preoperative and postoperative magnetic resonance imaging (MRI); (iii) having undergone head computed tomography (CT) after SEEG implantation; and (iv) having a postoperative follow-up duration of ≥2 years. Surgical outcomes were determined by epilepsy specialists based on follow-up findings, with a favorable outcome defined as ILAE Grade I according to the International League Against Epilepsy (ILAE) criteria (Wieser et al., 2001). Patients with incomplete clinical data or those who did not proceed with surgical treatment were excluded.Detailed participant information is listed in Table 1. Written informed consent was obtained from all participants. This study was approved by the Ethics Committee of the Second Hospital of Lanzhou University (Approval No.: 2023A-765).

**Table 1:** Patient demographics and clinical information.

**ID**	**Sex**	**Surgery age (Years)**	**Onset age (Years)**	**MRI**	**Seizures**	**Electrodes (SEEG)**	**Contacts (SEEG)**	**Surgical region**	**Therapy**	**Engel**
P01	M	31	27	P	5	8	94	RH, RA	R	E I
P02	M	23	3	P	4	9	118	RP, RT	T & R	E I
P03	F	12	11	N	5	10	140	RI	T	E I
P04	F	28	12	P	5	8	109	LT	R	E I
P05	M	36	18	P	2	8	122	LIFG, LMFG, LOFG	R	E I
P06	M	23	16	P	3	9	96	RT	T & R	E I
P07	M	15	3	P	3	7	74	RF	T	E I
P08	M	17	12	P	6	12	114	LF, LP	T & R	E I
P09	M	30	25	P	2	9	124	LT, LH	T & R	E I
P10	F	26	6	P	6	11	164	RP, RT	T	E I
P11	M	20	/	P	3	8	108	LP	R	E I
P12	F	16	13	P	2	8	105	RH, RA	T & R	E I
P13	M	12	7	P	4	8	124	RO	T & R	E I
P14	M	22	20	P	5	9	127	RFO, RA, RACS	T	E I
P15	F	35	16	P	5	9	112	LT	T	E I
P16	M	28	4	P	3	9	119	LT, LH	T & R	E I
P17	F	31	5	P	2	8	87	LPG	T	E I
P18	F	17	2	P	5	8	92	RF	T & R	E I
P19	F	26	7	N	3	8	108	RPO, RCO, RI	T	E I
P20	M	37	35	P	3	8	118	RT	T	E I
P21	F	20	18	P	4	8	114	RPOTJ	T	E I
P22	M	18	10	P	3	10	129	RP, RPCG	T	E I
P23	F	31	21	P	2	11	150	ROFG, RTP,RH	T & R	E I
P24	M	26	1	P	5	7	84	RT, RF	R	E I
P25	M	22	9	P	3	9	125	LHS	R	E I

Sex: M, male; F, female; MRI: N, negative; non-lesional patient, P, positive; lesional patient, / = information missing.

Surgical region (i.e., clinically annotated EZ): LF, Left frontal lobe; LHS, Left hemisphere; RPOTJ, Right parieto-occipital-temporal junction; LT, Left temporal lobe; RP, Right parietal lobe; RPCG, Right posterior cingulate gyrus; RFO, Right frontal operculum; RAI, Right anterior insula; RACS, Right anterior circular sulcus; RT, Right temporal lobe; LH, Left hippocampus; RF, Right frontal lobe; RO, Right occipital lobe; RI, Right insula; LPG, Left precentral gyrus; RPO, Right parietal operculum; RCO, Right central operculum; ROFG, Right orbital frontal gyrus; RTP, Right temporal pole; RH, Right hippocampus; RA, Right amygdala; LP, Left parietal lobe; LIFG, Left inferior frontal gyrus; LMFG, Left middle frontal gyrus; LOFG, Left orbital frontal gyrus.

Therapy: T, thermal coagulation; R, surgical resection.

### 2.2 EEG recordings and processing

Both SEEG and scalp EEG recordings were acquired using a Nihon Kohden EEG-1200C system. Scalp EEG electrodes were placed according to the international 10–20 system, with data recorded at a sampling rate of 500 Hz. SEEG data were sampled at 2,000 Hz. The number of implanted SEEG electrodes per patient ranged from 7 to 12. Each electrode consisted of 8–16 contacts, with a contact length of 2 mm, a diameter of 0.8 mm, and an inter-contact spacing of 1.5 mm. Each contact recorded neural activity, corresponding to a single SEEG channel. On average, 113 ± 20 (mean ± SD) SEEG channels per patient were included in the final analysis. SEEG contacts were classified as belonging to the EZ or NEZ based on the clinically defined surgical resection area. In this study, EEG preprocessing was completed using the EEGLAB and Brainstorm toolboxes in MATLAB (R2022b, MathWorks, Inc., USA) (Tadel et al., 2011). The preprocessing steps included: (1) Conduct band-pass filtering of EEG data between 0.5 and 300 Hz using a 4th-order Butterworth filter; (2) Perform notch filtering at 50 Hz and its harmonics, with a stopband of 2 Hz, to eliminate power-line interference; (3) Exclude “bad channels” that are damaged, excessively noisy, or contaminated with artifacts from the dataset; (4) Implement Independent Component Analysis (ICA) using the EEGLAB toolbox to remove artifacts such as eye movements and muscle activity from EEG signals; (5) Carry out band-pass filtering on EEG data to retain the delta (0.5–4 Hz), theta (4–8 Hz), alpha (8–13 Hz), beta (13–30 Hz), gamma (30–80 Hz) frequency bands, and high-frequency oscillations (HF, >80Hz). Across the 25 patients, seizure durations (defined as the interval from onset to termination) ranged from 20 to 120 seconds. For each preoperatively acquired EEG and SEEG recording, we extracted 10-second epochs from both the mid-ictal and interictal periods for brain network analysis.The mid-ictal epoch was selected based on its sustained duration and ease of clinical identification. Evidence indicates that the epileptic network undergoes dynamic reconfiguration during this stage; specifically, compared to the preseizure state, functional connectivity within the SOZ has been demonstrated to significantly strengthen and spatially tighten (Khambhati et al., 2015). Additionally, the global correlation structure in this phase presents unique generic changes independent of the anatomical seizure onset location, thereby enabling reliable identification of the network's dynamic evolution process (Schindler et al., 2007). The interictal EEG was chosen because the EEG signals in this phase are characterized by prominent stability and minimal artifact contamination. Furthermore, the interictal EEG network shows significant differences from healthy brain networks, allowing for the effective capture of the aberrant intrinsic dynamics of the epileptogenic network (Makaram et al., 2025; Carboni et al., 2020; Jiang et al., 2022).

### 2.3 EEG source imaging

In recent years, ESI has been proven to be effective in epileptic focus localization (Ye et al., 2021). From a mathematical perspective, EEG can be regarded as a linear combination of neural source activities. Solving for the electrical signals generated on the scalp when neural currents are known is referred to as the forward problem of EEG. In contrast, ESI is a process of solving the inverse problem—it non-invasively estimates the neural electrical activity underlying EEG by counteracting the effects of volume conduction or field propagation, and under anatomical and physiological constraints (Feng et al., 2025; He et al., 2018). In our study, 16-channel scalp EEG data were used, which is the most widely adopted configuration in clinical practice. We used Standardized Low-Resolution Electromagnetic Tomography (sLORETA) as the method for ESI, and this method has the advantages of small errors, stable performance, and high computational feasibility (Samuelsson et al., 2021; Mikulan et al., 2020). In the sLORETA algorithm, scalp EEG signals are modeled as


Φ=KJ+c1 (1)

Where $\Phi \in {\mathbb{R}}^{N_{E}\times 1}$is a vector containing scalp electric potentials measured at *N*_*E*_ cephalic electrodes, the vector $J\in {\mathbb{R}}^{\left(3N_{V}\right)\times 1}$ contains the primary (impressed) current densities of each of the *N*_*V*_ intracerebral voxels, and is defined as $J={\left(J_{1}^{\text{T}},J_{2}^{\text{T}},\ldots ,J_{N_{V}}^{\text{T}}\right)}^{\text{T}}$. At the *l*^th^ voxel, $J_{l}^{\text{T}}=\left(J_{l}^{x},J_{l}^{y},J_{l}^{z}\right)$ contains the three unknown dipole moments. The lead field $K\in {\mathbb{R}}^{N_{E}\times 3N_{V}}$ is defined as


$$K=\left[\begin{array}{cccc} k_{1,1} & k_{1,2} & \ldots & k_{1,N_{V}}\\ k_{2,1} & k_{2,2} & \ldots & k_{2,N_{V}}\\ \vdots & \vdots & \ddots & \vdots \\ k_{N_{E},1} & k_{N_{E},2} & \ldots & k_{N_{E},N_{V}} \end{array}\right]$$ (2)

with $k_{i,l}\in {\mathbb{R}}^{1\times 3}$, for *i* = 1, …, *N*_*E*_, and for *l* = 1, …, *N*_*V*_. Note that $k_{i,l}=\left(k_{i,l}^{x},k_{i,l}^{y},k_{i,l}^{z}\right)$ denotes the scalp electric potentials measured at the *i*-th electrode when there are unit-strength dipoles in the *x*, *y*, and *z* directions at the *l*-th voxel. c is an arbitrary constant, and $1\in {\mathbb{R}}^{N_{E}\times 1}$ is a vector of ones. The parameter c allows any reference to be used for the lead field and the measurement process. For EEG source localization, the aim is to estimate J, the primary current density. The function of interest is


$$F=||\Phi -KJ-c1|{|}^{2}+\alpha ||J|{|}^{2}$$ (3)

where α≥0 is a regularization parameter. This function is minimized with respect to *J* and *c*, for given *K*, Φ, and α.


$$Ĵ=\underset{J,c}{\min}F=\underset{J,c}{\min}\left(||\Phi -KJ-c1|{|}^{2}+\alpha ||J|{|}^{2}\right)$$ (4)

Finally, Ĵ is further scaled to become the source estimation of sLORETA. The process was conducted using the Boundary Element Method (BEM) model and corrected through MATLAB's brainstorm toolbox (Tadel et al., 2011). Based on the brain regions defined by the Destrieux Atlas, the results were presented across 148 channels, comprising 74 brain regions for each hemisphere (left and right). Subsequently, we labeled the surgical area as the EZ and the rest as NEZ. Since the data are from patients with successful surgery, these annotations can be considered true and effective, with the number of EZ channels ranging from 5 to 31 per patient.

### 2.4 Construction of functional brain networks

Functional connectivity matrices were constructed for each frequency band. We defined nodes based on the recording modality: for EEG, nodes represented cortical sources derived from source imaging, whereas for SEEG, they corresponded to individual electrode contacts. Functional connectivity was then calculated to establish the edges connecting these nodes (Pascual-Marqui, 2002; He et al., 2019). In this study, we employed the Pearson correlation coefficient (PCC) to quantify intra-network connectivity. PCC is a method used to calculate non-directed coupling relationships between time series (Benesty et al., 2009; Chiarion et al., 2023). The resulting connectivity matrix characterized the linear relationships between brain regions, with each element representing the functional connection strength between the corresponding nodes. Subsequently, unweighted, undirected binary networks were constructed by applying a fixed absolute threshold of 0.7 (*T* = 0.7). Connections with correlation values below this threshold were discarded (set to 0), while those above were retained (set to 1), ensuring that only strong functional couplings were analyzed. Previous studies indicate that in neurophysiological data, weak connections often reflect spurious correlations caused by volume conduction. Consequently, a relatively high threshold is necessitated to filter out these artifacts and isolate strong connections associated with epilepsy (Rubinov and Sporns, 2010). Conversely, an excessively high threshold may result in an overly sparse network, leading to the loss of true functional edges (Kramer et al., 2008). To address this issue, we conducted a sensitivity analysis by re-evaluating network metrics across thresholds of 0.6, 0.7, and 0.8. The results demonstrated that the core topological differences between the EZ and NEZ remained statistically robust across this range (see Supplementary materials). Although minor variations in significance levels were observed (e.g., in full-band EEG), the directionality and trends of the topological alterations remained consistent. This confirms that our findings reflect stable pathophysiological mechanisms of the epileptogenic network rather than methodological artifacts contingent upon a single threshold. Ultimately, we selected 0.7 as the threshold to strike a balance between noise exclusion and the preservation of meaningful network structures.

### 2.5 Graph theoretical analysis

Following the construction of epileptogenic networks for patients with DRE, we utilized the Brain Connectivity Toolbox (BCT) in MATLAB to compute network topological properties. Specifically, four key graph metrics were extracted for each node to characterize its functional importance within the network (Table 2).

**Table 2:** Local network features.

**Measure**	**Mathematical definition**	**Significance**
Betweenness centrality (BC)	$BC\left(i\right)=\underset{\begin{array}{c} s\neq i\neq t \end{array}}{\sum }\frac{\sigma_{st}\left(i\right)}{\sigma_{st}}$	Quantify a node's role as an information “bridge” within the network (Rubinov and Sporns, 2010).
Clustering coefficient (C)	$C_{i}=\frac{2e_{i}}{k_{i}\left(k_{i}-1\right)}$	Measure the connectivity density among a node's immediate neighbors (Rubinov and Sporns, 2010).
Local efficiency (LE)	$LE=\frac{1}{N}\underset{i\in G}{\sum }GE_{i}$	Evaluate the efficiency of local information transmission when the node is removed (Latora and Marchiori, 2001).
Node degree (ND)	$ND_{i}=\overset{N}{\underset{j=1}{\sum }}a_{ij}$	Reflect the relative importance of a node in the network (Rubinov and Sporns, 2010).

To mitigate the influence of variations in node count on local network characteristics, topological metrics were first computed for the entire network. Subsequently, nodal metrics corresponding to the EZ and NEZ were extracted based on clinical labels across seven frequency bands: full-band, delta (δ), theta (θ), alpha (α), beta (β), gamma (γ), and the high-frequency band. This yielded a total of 28 features (7 frequency bands × 4 network metrics), establishing a foundation for subsequent statistical comparison and machine learning classification.

### 2.6 Machine learning classification

To distinguish between EZ and NEZ, we employed five machine learning classifiers: Random Forest (RF), Support Vector Machine (SVM), Gradient Boosting Machine (GBM), K-Nearest Neighbors (KNN), and Logistic Regression (LR). The analysis was performed on a unified feature matrix integrating 28 topological features, with strictly balanced class distributions (1:1) across four datasets (scalp EEG and SEEG, both ictal and interictal). To address the sensitivity of distance-based and linear algorithms to feature magnitudes, we constructed a processing pipeline where a Z-score normalization (StandardScaler) was applied specifically for SVM, KNN, and LR. Crucially, this normalization was embedded within the cross-validation loop, ensuring that scaling statistics were derived solely from the training folds to prevent data leakage, while tree-based models (RF and GBM) were trained on the original feature space.

To guarantee robust model generalizability and avoid overfitting, we implemented a nested patient-level cross-validation strategy. The outer loop utilized a Group K-Fold (*k* = 5) partition based on unique patient identifiers, ensuring that all recordings from a specific patient were held out exclusively for testing in each iteration. Within each training fold of the outer loop, we performed an inner 3-fold cross-validation to execute a systematic Grid Search for hyperparameter optimization. Instead of relying on fixed default settings, hyperparameters were dynamically tuned from defined search spaces (e.g., KNN neighbors *k*∈{3, 5, …, 11}; RF estimators ∈{50, 100, 200} and max depth ∈{None, 10, 20}; SVM *C*∈{0.1, 1, 10}). The configuration yielding the highest AUC in the inner loop was then selected to train the final model for the outer test fold.

Model performance was comprehensively evaluated using metrics derived from the confusion matrix and probability scores, including Accuracy, Precision, Sensitivity, Specificity, and the Area Under the ROC Curve (AUC-ROC). By strictly adhering to patient-level stratification in both the outer evaluation loop and inner tuning loop, this protocol effectively prevented subject-specific data leakage, ensuring that the reported results reflect the models' true capacity to generalize to unseen subjects.

## 3 Results

### 3.1 Statistical analysis results

**Figure 2:**
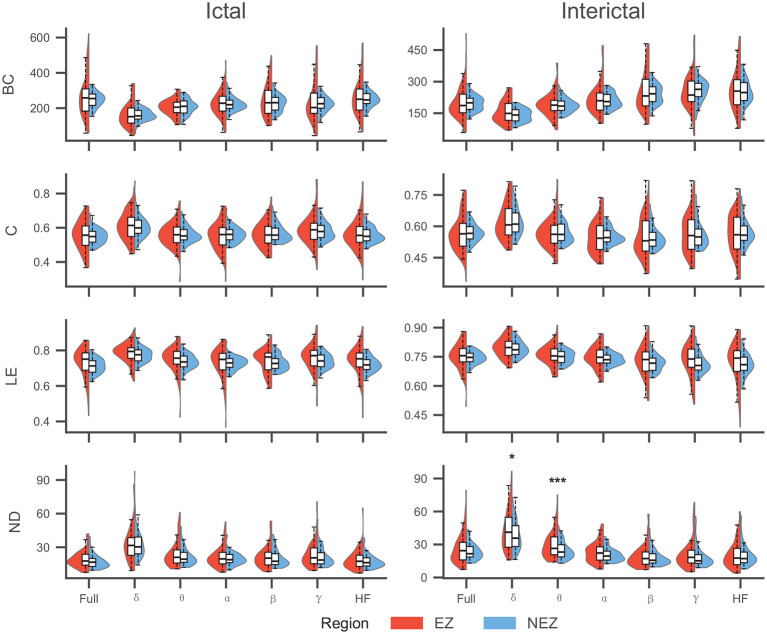
Comparison of topological features between EZ and NEZ in EEG. ND exhibited significant differences (NEZ > EZ) exclusively in the interictal low-frequency bands. No significant differences were observed for the remaining metrics across any frequency bands or states (^*^: <0.05,^***^: <0.001).

**Figure 3:**
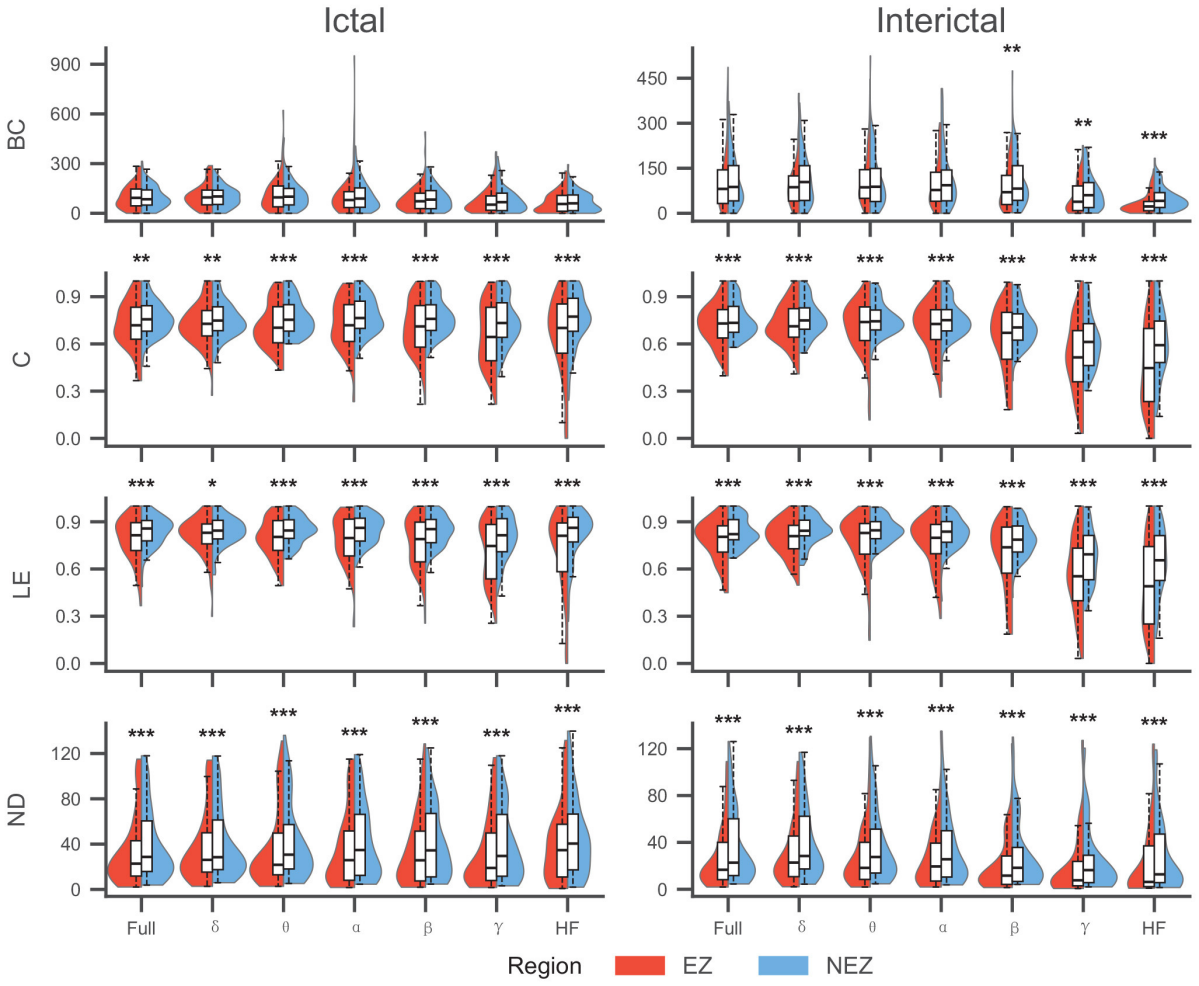
Comparison of Topological Features between EZ and NEZ in SEEG. C, LE, and ND showed significant differences (NEZ > EZ) across all frequency bands and states. However, for BC, significant differences (NEZ > EZ) were restricted to the interictal high-frequency bands (^*^: <0.05,^**^: <0.01,^***^: <0.001).

To identify topological features with discriminative capability between the EZ and NEZ, we conducted paired Wilcoxon signed-rank tests on the local network metrics derived from both EEG (ictal and interictal) and SEEG (ictal and interictal) recordings across all frequency bands. The Benjamini-Hochberg (BH) procedure was applied to correct for multiple comparisons. Comparisons were performed in a paired manner between the EZ and NEZ within each individual sample. The results are presented in Table 3, Figures 2, 3.

**Table 3:** *P*-values of paired Wilcoxon test for local network features of EZ and NEZ under different frequency bands.

	**Feature**	**Full**	**Delta**	**Theta**	**Alpha**	**Beta**	**Gamma**	**HF**
**EEG ictal**	**BC**	0.864	0.948	0.846	0.948	0.554	0.846	0.948
	**C**	0.846	0.840	0.948	0.840	0.701	0.840	0.948
	**ND**	0.204	0.948	0.512	0.645	0.512	0.216	0.554
	**LE**	0.204	0.204	0.216	0.701	0.512	0.204	0.204
**EEG interictal**	**BC**	0.869	0.105	0.944	0.944	0.827	0.827	0.795
	**C**	0.944	0.827	0.869	0.795	0.944	0.869	0.975
	**ND**	0.231	0.014	9.50 × 10^−4^	0.087	0.438	0.231	0.694
	**LE**	0.352	0.297	0.231	0.438	0.352	0.231	0.391
**SEEG ictal**	**BC**	0.871	0.833	0.715	0.465	0.247	0.568	0.568
	**C**	0.001	0.006	2.41 × 10^−7^	1.43 × 10^−5^	7.91 × 10^−8^	2.72 × 10^−8^	5.61 × 10^−7^
	**ND**	1.20 × 10^−6^	1.18 × 10^−4^	2.36 × 10^−7^	4.28 × 10^−8^	4.28 × 10^−8^	4.28 × 10^−8^	6.55 × 10^−4^
	**LE**	9.60 × 10^−5^	0.011	2.39 × 10^−7^	1.34 × 10^−6^	2.27 × 10^−8^	7.66 × 10^−9^	2.36 × 10^−7^
**SEEG interictal**	**BC**	0.279	0.064	0.777	0.236	0.007	0.002	2.00 × 10^−7^
	**C**	1.11 × 10^−4^	1.43 × 10^−5^	8.62 × 10^−5^	1.19 × 10^−4^	4.45 × 10^−6^	8.59 × 10^−7^	4.60 × 10^−8^
	**ND**	2.85 × 10^−10^	6.15 × 10^−9^	8.35 × 10^−10^	9.13 × 10^−7^	3.31 × 10^−10^	2.85 × 10^−10^	1.02 × 10^−9^
	**LE**	6.94 × 10^−7^	2.23 × 10^−8^	2.20 × 10^−6^	5.36 × 10^−6^	2.00 × 10^−7^	8.86 × 10^−8^	1.54 × 10^−8^

The statistical analysis, adjusted for multiple comparisons using the BH procedure, revealed distinct patterns of topological alterations between the EZ and NEZ across different recording modalities, frequency bands. A marked disparity was observed between intracranial and scalp EEG recordings. SEEG demonstrated superior discriminative power, yielding highly significant differences (*P* < 0.001) across the majority of frequency bands and topological features. In contrast, scalp EEG showed limited sensitivity, with most features failing to reach statistical significance (*P*>0.05) in distinguishing EZ from NEZ, particularly during the ictal state.

In the SEEG datasets, the discriminative performance varied by feature type and frequency band. For Clustering Coefficient, Degree, and Local Efficiency, consistent and statistically significant differences (*P* < 0.01 or *P* < 0.001) were observed across nearly all frequency bands—ranging from Delta to HF—in both ictal and interictal states. This suggests that the local segregation and centrality of the EZ network undergo fundamental alterations regardless of the frequency component analyzed.The Betweenness Centrality showed a strong frequency dependence. During the interictal state, BC was significantly different only in the higher frequency bands (β, γ, and HF, *P* < 0.01), while remaining non-significant in lower frequencies (δ to α). During the ictal state, BC failed to show significant differences across most bands.

For scalp EEG, topological distinctions were observed primarily in the interictal phase. Specifically, significant differences were found for Nodal Degree within the low-frequency bands (δ, *P* = 0.014; θ, *P* < 0.001), whereas other metrics (BC, C, LE) showed no significant group differences. In the mid-ictal state, paired statistical comparisons of individual topological features did not reach the significance threshold (*P*>0.05) across frequency bands, presenting a distinct statistical profile compared to the invasive recordings.

### 3.2 Machine learning results

The classification performance of the machine learning models was evaluated across three distinct data categories: scalp EEG, raw SEEG, and preprocessed SEEG (with log-transformation and Z-score normalization). Detailed performance metrics are summarized in Table 4.

**Table 4:** Classification performance for EZ and NEZ.

**Dataset**	**Model**	**AUC**	**Accuracy**	**Sensitivity**	**Specificity**	**Precision**	**F1-score**
**EEG ictal**	SVM*	**0.924**	**0.865**	**0.889**	**0.841**	**0.848**	**0.868**
	RF	0.903	0.817	0.794	0.841	0.833	0.813
	GBM	0.861	0.762	0.714	0.810	0.789	0.750
	KNN	0.789	0.603	0.222	0.984	0.933	0.359
	LR	0.550	0.579	0.429	0.730	0.614	0.505
**EEG interictal**	SVM*	**0.927**	**0.857**	**0.857**	**0.857**	**0.857**	**0.857**
	RF	0.888	0.794	0.730	0.857	0.836	0.780
	GBM	0.862	0.794	0.714	0.873	0.849	0.776
	KNN	0.772	0.516	0.048	0.984	0.750	0.090
	LR	0.593	0.635	0.524	0.746	0.673	0.589
**SEEG ictal**	SVM	0.670	0.614	0.675	0.554	0.602	0.636
	RF*	**0.680**	**0.620**	**0.542**	**0.699**	**0.643**	**0.588**
	GBM	0.649	0.584	0.602	0.566	0.581	0.592
	KNN	0.625	0.584	0.301	0.867	0.694	0.420
	LR	0.600	0.602	0.482	0.723	0.635	0.548
**SEEG interictal**	SVM	0.587	0.620	0.735	0.506	0.598	0.659
	RF	0.692	0.657	0.590	0.723	0.681	0.632
	GBM	0.602	0.584	0.566	0.602	0.588	0.577
	KNN*	**0.708**	**0.669**	**0.494**	**0.843**	**0.759**	**0.599**
	LR	0.680	0.651	0.771	0.530	0.621	0.688
**SEEG ictal (Log+Z)**	SVM	0.799	0.717	0.711	0.723	0.720	0.715
	RF*	**0.818**	**0.741**	**0.747**	**0.735**	**0.738**	**0.743**
	GBM	0.745	0.705	0.699	0.711	0.707	0.703
	KNN	0.763	0.687	0.542	0.831	0.763	0.634
	LR	0.806	0.729	0.723	0.735	0.732	0.727
**SEEG interictal (Log+Z)**	SVM*	**0.872**	**0.819**	**0.831**	**0.807**	**0.812**	**0.821**
	RF	0.868	0.795	0.831	0.759	0.775	0.802
	GBM	0.837	0.789	0.771	0.807	0.800	0.785
	KNN	0.844	0.795	0.735	0.855	0.836	0.782
	LR	0.864	0.813	0.819	0.807	0.810	0.814

^*^is the best model.

**Figure 4:**
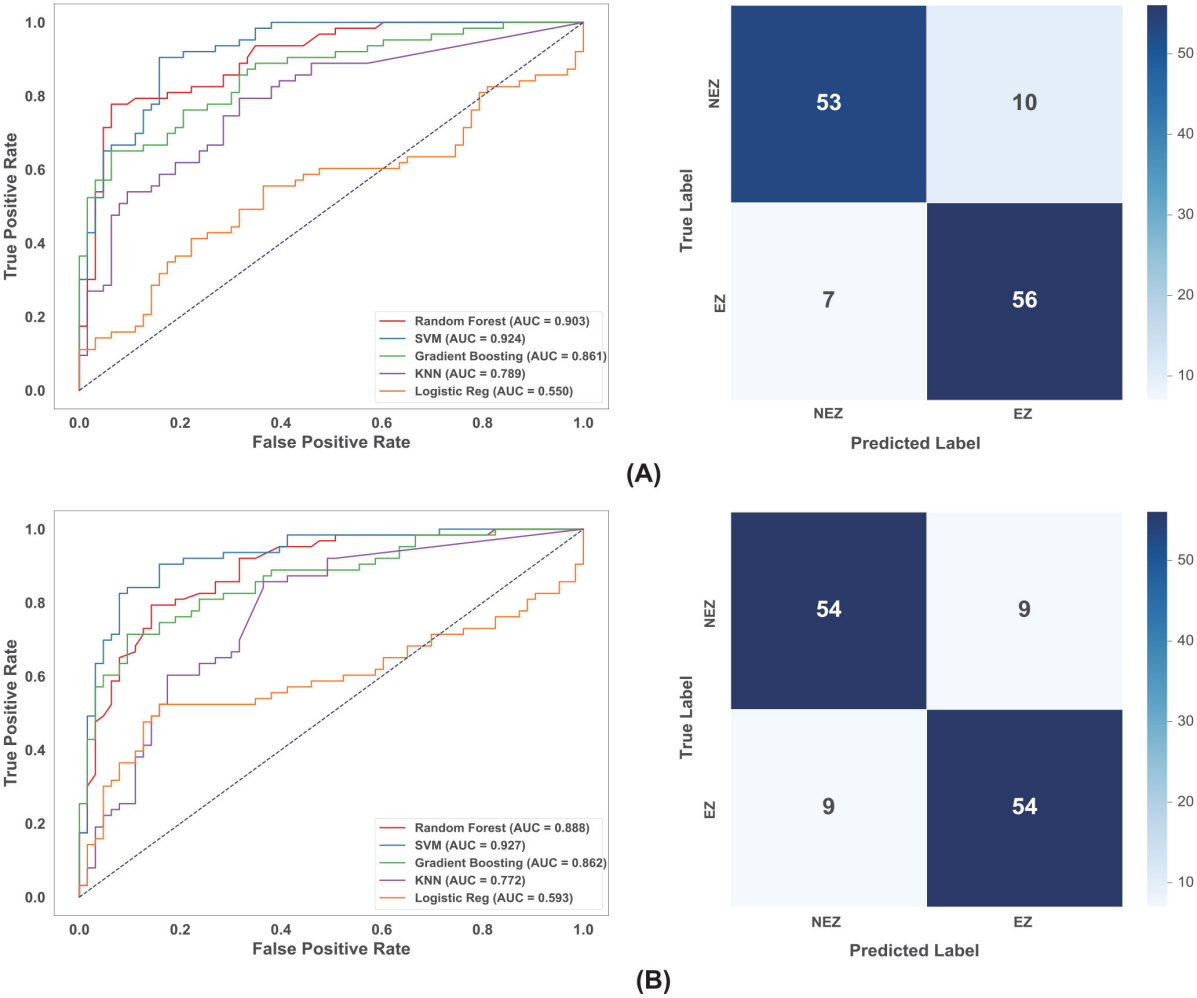
ROC curves of multiple models and confusion matrices of SVM for EEG modality in different state. **(A)** Ictal state. **(B)** Interictal state.

Models trained on graph theoretical features derived from scalp EEG source localization demonstrated the highest overall performance. Among the evaluated classifiers, SVM emerged as the superior model, achieving the highest AUC in both the ictal (0.924) and interictal (0.927) states (as shown in Figure 4). Tree-based ensemble methods, such as RF and GBM, also demonstrated strong performance with AUCs exceeding 0.86. In contrast, simpler distance-based and linear models, specifically KNN and LR, underperformed significantly; for instance, KNN yielded a sensitivity of only 22.2% in the ictal state, and LR achieved an AUC of just 0.550. This marked performance gap suggests that the topological distinction between EZ and NEZ in scalp EEG likely resides in a high-dimensional, non-linearly separable feature space, where SVM's kernel mapping capabilities provide a decisive advantage over algorithms relying on linear decision boundaries or simple Euclidean distances.

**Figure 5:**
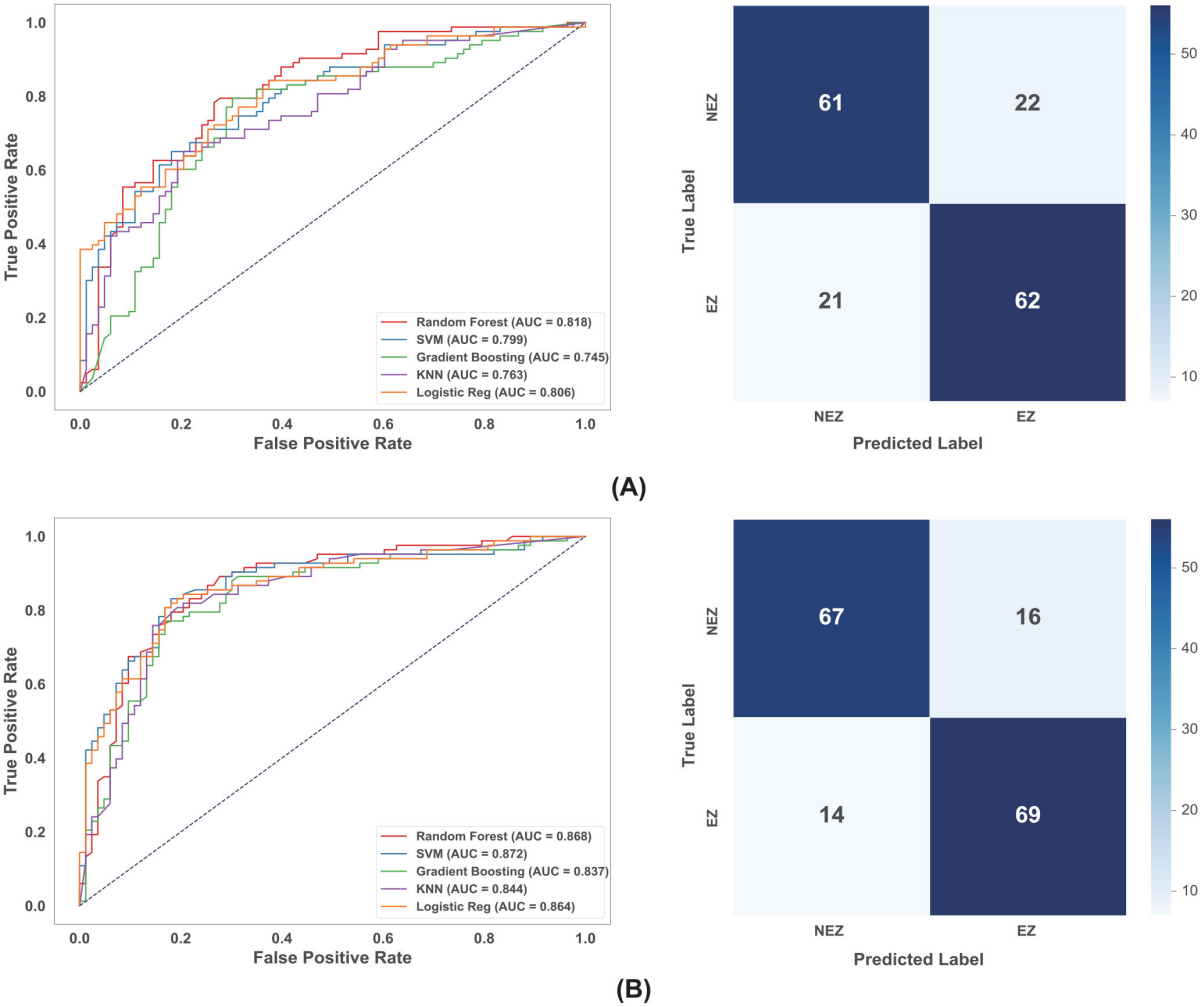
ROC curves of multiple models and confusion matrices of RF (ictal) or SVM (interictal) for SEEG modality after log-transformation and Z-score normalization in different state. **(A)** Ictal state. **(B)** Interictal state.

For the SEEG modality, the analysis revealed a critical dependency on data preprocessing. Using raw SEEG datasets, classification performance was generally suboptimal across all algorithms (AUC range: 0.58–0.71), likely due to the high inter-subject variability and non-Gaussian distribution of the raw topological metrics. In this noisy environment, Random Forest and KNN showed relative resilience, attributable to RF's ensemble averaging and KNN's local decision boundaries, which are less sensitive to global distribution shifts than parametric models. However, the application of our proposed preprocessing pipeline—log-transformation followed by Z-score normalization—yielded a substantial and uniform performance improvement. This step proved critical for unlocking the potential of the algorithms, particularly for SVM and Logistic Regression, which were previously hampered by feature scaling issues. Notably, in the SEEG interictal state, the AUC of the SVM model surged from 0.587 on raw data to 0.872 after preprocessing, becoming the top-performing model, while Random Forest maintained its robustness to achieve the highest performance in the ictal state (AUC = 0.818) (as shown in Figure 5).

Comparing the algorithms overall, SVM demonstrated the highest overall potential, serving as the optimal choice for both scalp EEG and preprocessed SEEG data due to its ability to model complex, non-linear relationships. Random Forest exhibited the highest stability and robustness against noise, making it a reliable baseline model, particularly for raw or unscaled data where other models may fail. While scalp EEG features are robust enough for direct classification, SEEG features require specific preprocessing to mitigate inter-subject variability, after which the topological biomarkers proved to be highly diagnostic (AUC > 0.87) across different classifiers. From a dataset perspective, scalp EEG demonstrated comparable classification performance across both ictal and interictal states; in contrast, for SEEG, the interictal performance was superior to that of the ictal state.

**Figure 6:**
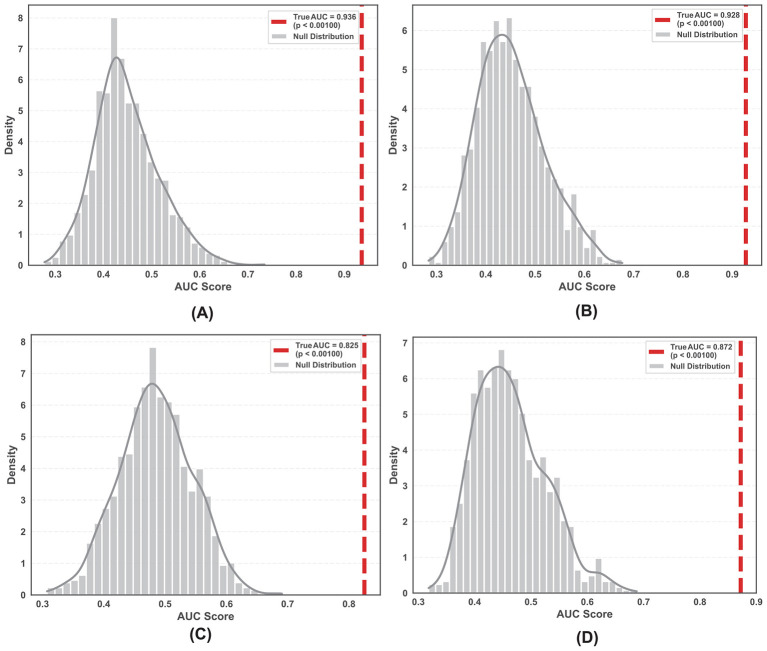
Permutation tests for the best-performing models in four datasets. **(A)** EEG ictal state. **(B)** EEG interictal state. **(C)** SEEG ictal state (after log-transformation and Z-score normalization). **(D)** SEEG interictal state (after log-transformation and Z-score normalization).

Permutation tests (*n* = 1,000) were conducted to validate the statistical significance of the best-performing models. The true AUC scores for all datasets—including scalp EEG, raw SEEG, and preprocessed SEEG—significantly exceeded the null distributions generated by random label shuffling. The *p*-values were <0.001 for the EEG and preprocessed SEEG datasets, confirming that the classification results are statistically significant and distinct from random chance (Figure 6).

## 4 Discussion

In this study, we employed a paired experimental design to evaluate the topological alterations of the EZ compared to the NEZ. By analyzing both SEEG and source-resolved scalp EEG, our analysis revealed a striking, modality-dependent divergence: the EZ manifests as a functionally isolated sink in SEEG but appears as a pathological hub in scalp EEG. This dissociation was consistent across both ictal and interictal states and provided robust discriminative features for our machine learning classifier.

### 4.1 SEEG perspective: structural and functional isolation

In SEEG recordings, the nodal metrics of the EZ—specifically Clustering Coefficient, Local Efficiency, and Node Degree—were significantly lower than those of the NEZ across the full frequency spectrum (*P* < 0.001). This finding aligns closely with the “Interictal Suppression Hypothesis (ISH)” recently established by Doss et al. (2024). The ISH posits that during the interictal state, the healthy brain network actively suppresses the EZ by limiting its outward connectivity to prevent seizure propagation, thereby rendering the EZ functionally isolated in the network topology.

However, local metrics alone cannot discern whether this isolation stems from a disconnection from the network or a disintegration within the node itself. To resolve this topological ambiguity, we conducted a targeted sub-network density analysis by partitioning the brain into EZ and NEZ modules. We defined “Internal Density” as the ratio of observed connections to possible connections within a module, reflecting its intrinsic cohesion, and “Interaction Density” as the connectivity strength bridging the two modules. This deconstruction revealed two distinct mechanistic components of the isolation. Similarly, paired Wilcoxon signed-rank tests and BH correction were performed on these metrics.

First, the isolation is rooted in an “Internal Structural Breakdown.” In the majority of frequency bands, the internal density of the EZ (*D*_*EZ*_) was significantly lower than that of the NEZ (*D*_*NEZ*_) (*P* < 0.05). This indicates that the EZ is not merely detached from the outside; rather, it is internally loose and fragmented. This finding is supported by recent structural connectivity studies, such as Yildirim et al. (2025) which demonstrated that the morphological substrates of epilepsy (e.g., Hippocampal Sclerosis) are associated with decreased structural integrity and connectivity due to local neuronal loss and gliosis. The physical depletion of neurons and synapses in the sclerotic or dysplastic tissue naturally precludes the formation of dense functional loops (Zhou et al., 2025).

Second, the brain maintains a “Modular Defense” mechanism (Makhoul et al., 2025). Crucially, our analysis revealed that the interaction density between regions (*D*_*EZ*-*NEZ*_) was consistently lower than the internal densities of both the EZ and NEZ (*P* < 0.01). This provides mesoscopic topological evidence for the suppression hypothesis proposed by Doss et al. (2024). The brain maintains a “functional fence” that actively restricts information flow between the pathological focus and the healthy connectome, manifest as minimal interaction density.This confirms that the EZ and NEZ function as two relatively independent modules. Both regions exhibit a preference for dense internal connectivity while maintaining sparse inter-regional connections, which further elucidates the phenomena observed in our local network metrics.

We also compared the internal and interaction density between the ictal and interictal periods to explore the dynamic mechanisms of the epileptic network. We observed that the ictal phase was accompanied by a widespread surge in network connectivity, yet exhibited significant heterogeneity across frequency bands and brain regions. Notably, despite the global increase in connectivity, the EZ and NEZ maintained a degree of functional segregation. In the beta, gamma and the high-frequency bands, connection densities—whether *D*_*EZ*_, *D*_*NEZ*_ or *D*_*EZ*-*NEZ*_—significantly increased compared to the interictal state (all *P* < 0.01). This dynamic shift mirrors the “Collapse of Suppressive Networks” described by Makhoul et al. (2025). According to this model, the transition to seizure occurs when the interictal inhibitory constraint fails, permitting the pathological hypersynchrony to break through the functional fence and spread globally.

However, despite this rise, the pattern of “EZ weaker than NEZ” remained robust in medium-to-high frequency bands (θ–γ) (*P* < 0.05). This suggests that while the EZ explodes upon the collapse of suppression, the recruited healthy tissue (NEZ) generates an even stronger “relative hypersynchrony,” forming a coherent “entrained alliance” that statistically overshadows the EZ.

A notable exception emerged in the Delta band. This was the only band where the EZ internal density (*D*_*EZ*_) showed a significant increase (*P* = 0.039) while the NEZ remained statistically stable. This selective enhancement allowed the EZ to catch up with the NEZ, resulting in no significant difference between their internal densities (*P* = 0.897) in the ictal state. We interpret this as the activation of Pathological Slow-Wave Resonance within the epileptogenic tissue. As demonstrated by Douw et al. (2010) focal epilepsy is characterized by significantly increased functional connectivity specifically in the Delta band localized to the pathological zone. Our findings suggest that during the transition to seizure, the EZ establishes a hypersynchronous state at its intrinsic pathological frequency (Delta), thereby serving a critical recruitment role.

### 4.2 Scalp EEG perspective: the “hub” topology in resting-state

In sharp contrast to the intracranial findings, source-resolved scalp EEG identified the EZ as a “pathological hub” with elevated connectivity metrics. This apparent contradiction—where the EZ is a hub in EEG but isolated in SEEG—reflects the interaction between the EZ's intrinsic dynamics and the physical properties of scalp recording.

Physiologically, the EZ is characterized by focal slowing (increased δ/θ activity) and local neuronal disorganization. As described by Vorwerk et al. (2014) the skull acts as a natural low-pass filter. This physical property selectively attenuates high-frequency signals while permitting the passage of low-frequency oscillations. Consequently, the chaotic high-frequency asynchronous activity within the EZ is spatially confined and largely undetectable at the scalp level. In contrast, slow waves are less attenuated by the resistive properties of the skull and possess higher spatial coherence, enabling them to penetrate multilayered tissues and propagate extensively via volume conduction. Thus, scalp EEG captures a “physically filtered” signal; while this process results in the loss of high-frequency information, it preserves the spatially coherent slow activity, resulting in the EZ exhibiting greater consistency compared to SEEG.

The specific limitations of source-resolved EEG inadvertently accentuate the topological role of the EZ as a connectivity hub. Solving the EEG inverse problem with a sparse 16-channel montage necessitates robust spatial smoothness constraints, inevitably introducing a significant Point Spread Function (PSF). As emphasized by Schoffelen and Gross, this results in “signal leakage” or “field spread,” where the activity of a single underlying source is smeared across neighboring voxels, generating spurious correlations Schoffelen and Gross, (2009).

Crucially, this leakage effect is not spatially uniform; rather, it is amplitude- and SNR-dependent. High-amplitude signals with high signal-to-noise ratios (SNR) precipitate more severe signal leakage. The EZ acts as a powerful generator of high-amplitude pathological slowing (e.g., focal δ/θ oscillations) and interictal spikes, possessing an SNR significantly superior to the background activity of the NEZ. Due to the linearity of the inverse operator, this high-energy signal from the EZ “leaks” more intensely into its surroundings, creating a dense, spatially coherent “halo” of mathematically induced high connectivity. In contrast, the lower-amplitude, stochastic background activity within the NEZ produces weaker leakage that is less likely to coalesce into statistically significant connectivity clusters. Consequently, the “hub” topology observed at the EZ is largely a methodological artifact: it represents the spatial smearing of a dominant pathological driver, differentiating it from the weaker background network of the NEZ.

While this process filters out the local chaotic dynamics (visible in SEEG), it inadvertently highlights the EZ on the macroscopic scale, creating the distinct feature space that enabled our classifier to distinguish the EZ from the whole-brain network effectively.

### 4.3 Validation of topological biomarkers via machine learning

To validate the diagnostic robustness and discriminative power of the identified topological signatures at the individual level, we employed a machine learning framework as a verification tool. The superior classification performance (AUC > 0.92) achieved by SVM confirms that the “pathological hub” topology observed in scalp EEG is a highly consistent and distinguishable feature compared to the background network. This also suggests that the topological distinctions between the EZ and NEZ in scalp EEG likely reside within a high-dimensional, non-linearly separable feature space. It is precisely this characteristic that confers a decisive advantage upon Support Vector Machines (SVM) over algorithms relying on linear decision boundaries or simple Euclidean distances Cortes and Vapnik, (1995). The high sensitivity (>85%) suggests that, despite the presence of volume conduction effects, the characteristic electrical activity generated by the EZ paradoxically leverages this signal leakage to amplify the contrast with the NEZ. This enhancement allows the EZ to be reliably differentiated from the weaker background network.

**Figure 7:**
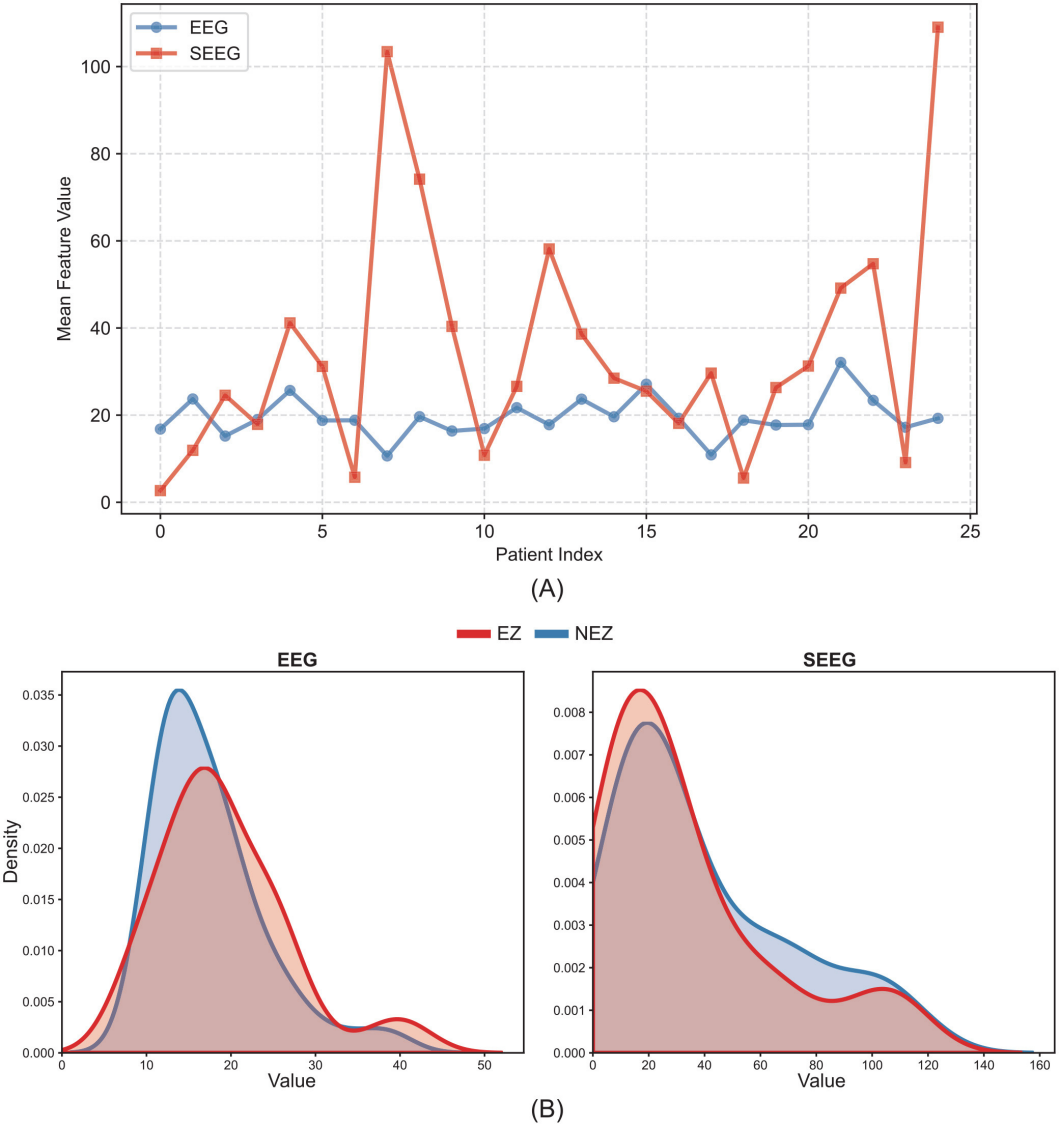
Line charts and KDE plots demonstrate the variability and distribution overlap of the data. **(A)** Multi-series line chart with markers of fullband node degree. **(B)** Kernel density estimation plot of fullband node degree.

In contrast, the classification performance using raw SEEG features was initially suboptimal (AUC 0.60–0.70), which is inconsistent with the significant topological differences previously observed between the EZ and NEZ at the SEEG level. We attribute this discrepancy to high inter-subject variability (Coefficient of Variation: SEEG 0.4 > EEG 0.16), arising from differences in baseline bioelectric activity, electrode implantation depths, and contact numbers (as shown in Figure 7). While paired tests controlled for individual differences within the same subject, the cross-subject learning task suffered from numerical overlap between EZ and NEZ features across individuals, obscuring the decision boundary. To address this, the introduction of Z-score and Log transformation proved pivotal, boosting the interictal SEEG AUC to 0.872. By applying log-transformation to correct the power-law distribution of nodal metrics and using Z-scores to highlight relative deviations at the individual level, we effectively minimized inter-subject heterogeneity. This approach converted absolute values into relative anomalies strictly within the training folds, enabling classifiers to detect the EZ with high precision.

Notably, our models performed comparably, or even slightly better, in the interictal state compared to the ictal state (EEG AUC: 0.927 vs. 0.924; SEEG-Zlog AUC: 0.872 vs. 0.818). This observation may be attributed to the dynamic nature of seizure evolution: the ictal state is characterized by the collapse of suppressive networks, facilitating the rapid breakthrough and widespread generalization of pathological activity (Makhoul et al., 2025). Conversely, the interictal state is effectively constrained by local suppression mechanisms, thereby preserving the distinct, focal topological signatures of the EZ with greater fidelity (Doss et al., 2024). Clinically, this is significant because traditional presurgical evaluation is contingent upon capturing seizures, which requires prolonged, costly, and uncomfortable monitoring. Our results demonstrate that the topological footprint of the EZ is a stable trait that persists interictally. This suggests that resting-state network analysis could serve as a rapid, seizure-free surrogate for EZ localization. Future Computer-Aided Diagnosis systems could utilize short-term interictal recordings to provide localization hypotheses, significantly reducing the clinical burden.

Our method demonstrates distinct advantages when compared to functional magnetic resonance imaging (fMRI) and positron emission tomography (PET). Although rs-fMRI detects EZ with high sensitivity (population mean 0.91), its clinical utility is often compromised by low specificity (population mean 0.09) (Boerwinkle et al., 2024), leading to a high rate of false positives. Similarly, PET imaging is costly and exhibits variable localization precision, with a recent meta-analysis reporting a pooled sensitivity of 0.81 and specificity of 0.71 (Niu et al., 2021). In contrast, our interictal EEG model achieved superior diagnostic performance, yielding an AUC > 0.92 with both sensitivity and specificity exceeding 0.85. This indicates that our topological approach provides a more accurate and specific delineation of EZ than metabolic or hemodynamic imaging. ESI covers the entire cortical surface, offering the distinct advantage of broad spatial coverage compared to the focal monitoring of SEEG. Consequently, as a non-invasive approach, the classification of ESI-based brain network features holds substantial promise for determining the approximate location of the EZ in presurgical evaluation.

### 4.4 Limitations

While this study elucidates the key topological features of the EZ within interictal networks and validates their translational potential, several limitations remain to be addressed in future work.

First, the spatial sampling density of the scalp EEG was relatively low. We utilized a standard clinical 16-channel montage. Although this configuration offers high clinical ubiquity and ease of generalization, its spatial resolution and source localization precision are inevitably limited compared to high-density EEG (HD-EEG, e.g., 128 or 256 channels). Sparse electrode coverage may result in insufficient capture of deep brain signals and increases the risk of signal leakage (point-spread effect) in the source space. However, our study focused on macroscopic network topology rather than millimeter-level anatomical localization. Furthermore, our machine learning results demonstrate that these topological features retain robust classification capabilities even at lower resolutions.

Second, the construction of the forward model relied on a standard brain template rather than individual MRI scans. While this approach enhances the generalizability of the method, neglecting individual variations in skull thickness and gyral anatomy may introduce source localization errors. Future studies incorporating individualized head models would likely further improve localization accuracy.

Finally, there are limitations regarding sample size and data types. Although we performed rigorous cross-validation at the single-subject level, the overall cohort size remains relatively small. Additionally, this study primarily focused on static network features during interictal and ictal periods, without deeply exploring the dynamic temporal evolution of the epileptic network.

## 5 Conclusion

In this study, we elucidated the distinct, modality-dependent network topological features of the EZ through a comparative analysis of EEG and SEEG. Our findings reveal a “micro-isolation vs. macro-hub” paradox: the EZ manifests as a functionally disconnected node at the mesoscopic SEEG scale, whereas it appears as a “pathological hub” with high connectivity strength at the macroscopic scalp EEG level.

Furthermore, our validation using machine learning confirmed that these topological signatures are robust and diagnostically valuable. The superior performance of the scalp EEG model highlights its utility for coarse localization and screening, while the SEEG analysis confirms that “relative isolation” is the primary feature for defining precise surgical boundaries. Importantly, we demonstrated that these topological footprints are stable traits that persist during the interictal state, challenging the traditional reliance on seizure capture.

Based on these findings, we propose a hierarchical operational framework: utilizing resting-state scalp EEG for initial hypothesis formulation, followed by SEEG analysis to precisely delineate the isolated epileptogenic tissue. This strategy offers a promising pathway for reducing the clinical burden of long-term monitoring and improving the precision of presurgical evaluation.

## Data Availability

The raw data supporting the conclusions of this article will be made available by the authors, without undue reservation.
