# Supplementary material for: Locating the epileptogenic zone for drug-resistant epilepsy through neuroelectrophysiological brain network topology
Source: Front Neurosci. 2026 Feb 11;20:1781032. doi: 10.3389/fnins.2026.1781032 (PMC12932511; doi:10.3389/fnins.2026.1781032)
Supplement: Supplementary file 1 [file Data_Sheet_1.pdf]

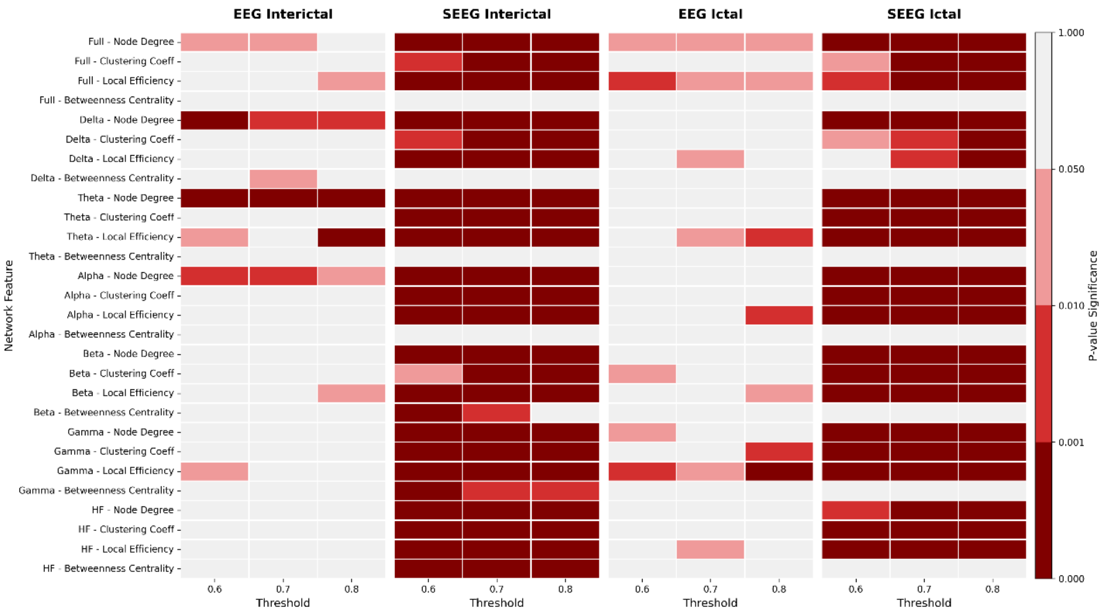

Supplementary Figure S1. Sensitivity analysis of topological differences between the Epileptogenic Zone (EZ) and Non-Epileptogenic Zone (NEZ) across varying network thresholds. The heatmap displays the statistical significance (*P*-values) of four network metrics (Node Degree, Clustering Coefficient, Local Efficiency, and Betweenness Centrality) across seven frequency bands (Full, Delta, Theta, Alpha, Beta, Gamma, HF). Columns represent the three thresholds tested (T=0.6, 0.7, 0.8). The analysis was performed separately for EEG Interictal, SEEG Interictal, EEG Ictal, and SEEG Ictal. **Key findings:** The core topological signatures—specifically the elevated Node Degree in low-frequency bands (Delta/Theta) for scalp EEG and the functional isolation (all features) for SEEG—exhibit consistent statistical significance across all thresholds, demonstrating the robustness of these biomarkers against parameter selection.

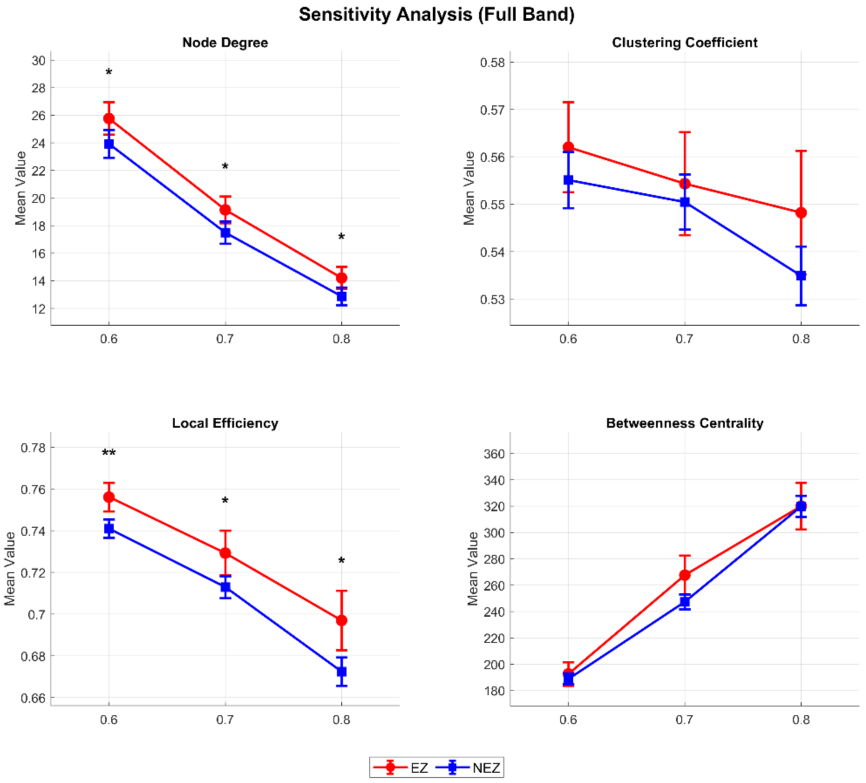
Sensitivity Analysis (Full Band)
Node Degree
Clustering Coefficient
Local Efficiency
Betweenness Centrality
EZ
NEZ

(A)

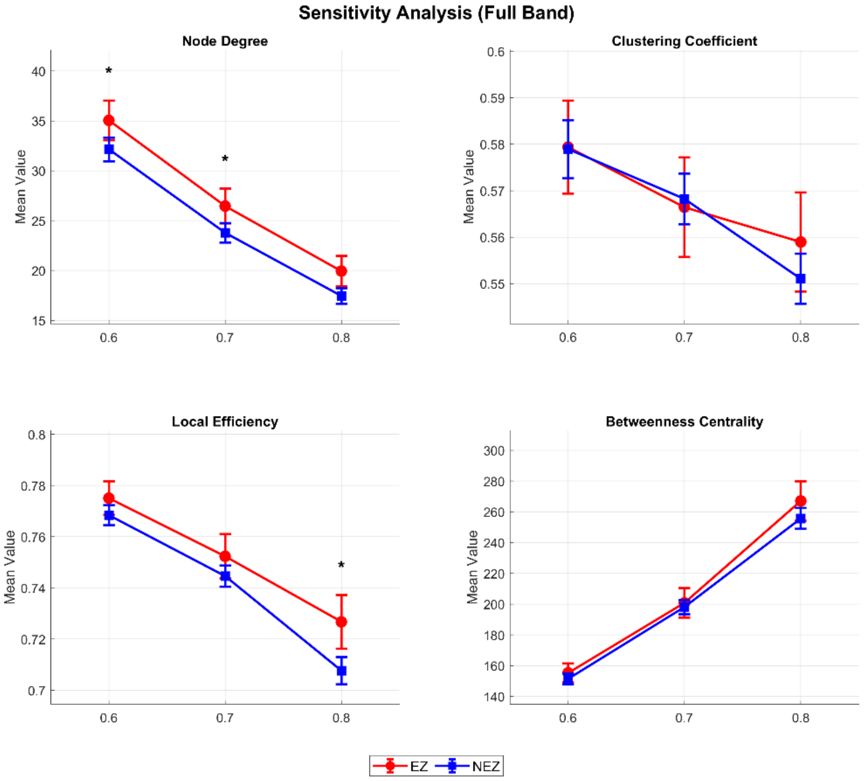
Sensitivity Analysis (Full Band)
Node Degree
Clustering Coefficient
Local Efficiency
Betweenness Centrality
EZ
NEZ

(B)

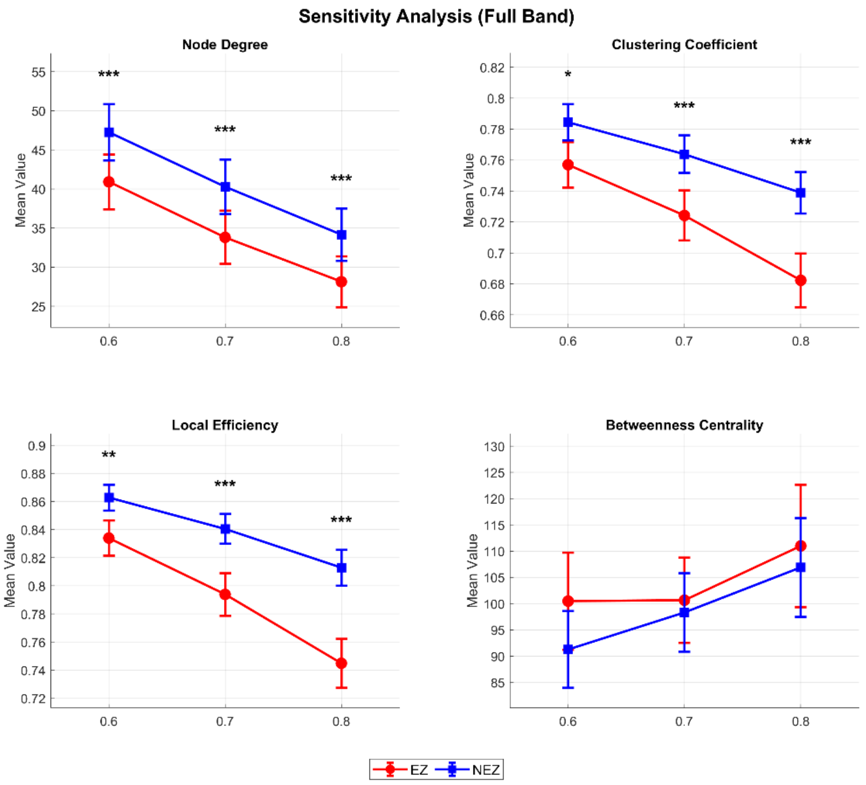

(C)

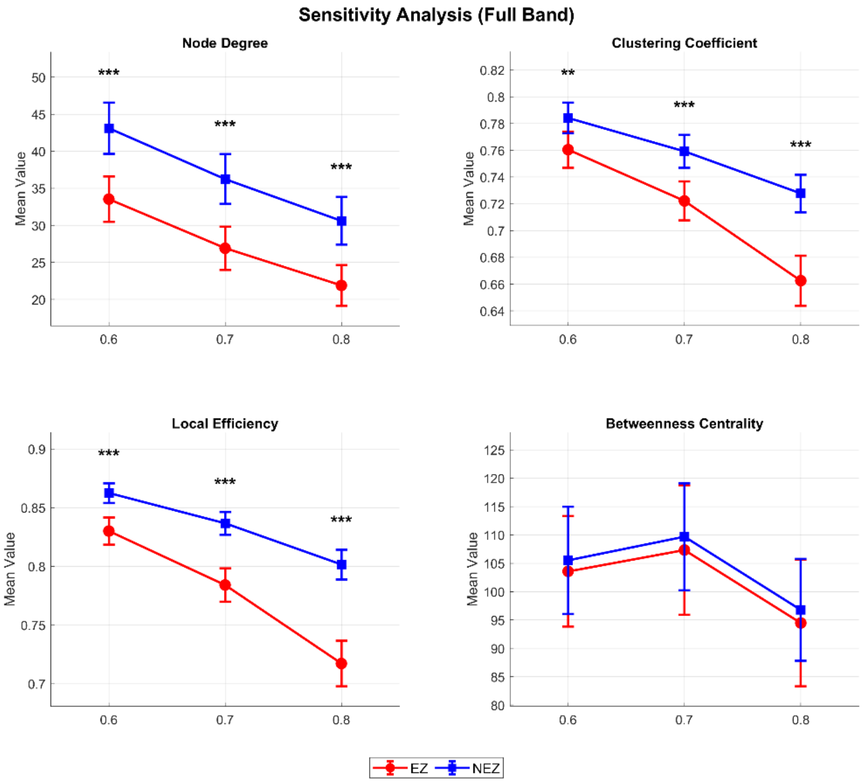

(D)

Supplementary Figure S2. Sensitivity analysis of topological network metrics in the full frequency band across varying thresholds. The line charts illustrate the trends of four key

graph-theoretical metrics—Node Degree, Clustering Coefficient, Local Efficiency, and Betweenness Centrality—derived from the EEG / SEEG recordings in the full frequency band. The analysis was performed across three different correlation thresholds ($T=0.6, 0.7, 0.8$). Data points indicate the mean value across all patients, and error bars represent the standard error of the mean (SEM). Statistical significance was assessed using paired Wilcoxon signed-rank tests ($*P < 0.05, **P < 0.01, ***P < 0.001$). **Key findings:** The significant differences between EZ and NEZ remained consistent across all thresholds for Node Degree, Clustering Coefficient, and Local Efficiency, demonstrating the robustness of these topological features against parameter selection. **(A) EEG Ictal (B) EEG Interictal (C) SEEG Ictal (D) SEEG Interictal**
